# Kyste hydatique orbitaire: cause non exceptionnelle d'exophtalmie au Maroc

**DOI:** 10.11604/pamj.2013.15.147.3167

**Published:** 2013-08-23

**Authors:** Hanan Handor, Moulay Zahid Bencherif

**Affiliations:** 1 Université Mohammed V Souissi, Service d'Ophtalmologie A de l'hôpital des spécialités, Centre hospitalier universitaire, Rabat, Maroc

**Keywords:** Kyste hydatique, orbite, exophtalmie, ophtalmoplégie, Hydatid cyst, eye socket, exophtalmia, ophthalmoplegia

## Images en médecine

Le kyste hydatique intra orbitaire est une entité clinique rare, qui touche le plus souvent l'enfant et l'adulte jeune vivant en milieu rural. Il est secondaire au développement au niveau de l'orbite du taenia *Echinococcus granulosus* dont l'hôte définitif est le chien et l'hôte intermédiaire est le mouton. Dans le cycle parasitaire l'Homme n'est qu'un hôte intermédiaire accidentel. Le Maroc est un pays d'endémie où l'hydatidose sévit encore et constitue une cause non exceptionnelle d'exophtalmie. A ce juste titre, nous rapportons l'observation d'un enfant âgé de 12 ans vivant à la campagne et qui a consulté aux urgences pour prise en charge d'une exophtalmie de l’œil droit d'installation progressive. L'examen à l'admission retrouve une exophtalmie non inflammatoire, indolore, non pulsatile et irréductible de l’œil droit avec un globe dévié vers le haut et une ophtalmoplégie complète. L'examen du fond d’æil était normal en dehors de la présence de quelques plis choroïdiens. Le scanner orbito-cérébral réalisé a mis en évidence au niveau de l'orbite droite une formation bien limitée de densité liquidienne refoulant le globe en avant et en haut. Cette formation ne prend pas le contraste à sa partie centrale mais le prend au niveau de la coque. L'abord chirurgical de l'orbite par orbitotomie antérieure a permis d'accoucher le kyste sans le rompre. Le kyste contenait un liquide eau de roche. L'examen anatomopathologique de la membrane proligère a confirmé le diagnostic de kyste hydatique orbitaire.

**Figure 1:**
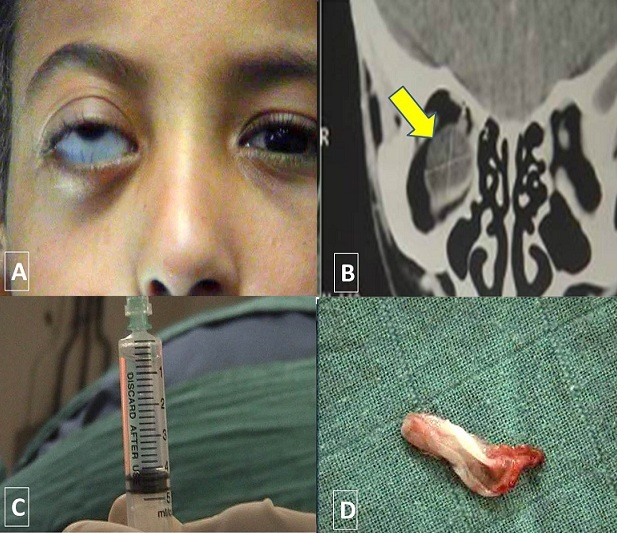
A): exophtalmie de l’œil droit avec un globe dévié vers le haut; B) TDM orbito cérébrale en coupe coronale objectivant une formation kystique de densité liquidienne au niveau de l'orbite droite (flèche); C) aspect eau de roche du liquide contenu dans le kyste; D) aspect macroscopique de la membrane proligère

